# Combinations of SERPINB5 gene polymorphisms and environmental factors are associated with oral cancer risks

**DOI:** 10.1371/journal.pone.0163369

**Published:** 2017-03-24

**Authors:** Hsiu-Ting Tsai, Ming-Ju Hsieh, Chiao-Wen Lin, Shih-Chi Su, Nae-Fang Miao, Shun-Fa Yang, Hui-Chuan Huang, Fu-Chih Lai, Yu-Fan Liu

**Affiliations:** 1 Accelerated Bachelor of Science in Nursing, College of Nursing, Taipei Medical University, Taipei, Taiwan; 2 School of Nursing, College of Nursing, Taipei Medical University, Taipei, Taiwan; 3 Institute of Medicine, Chung Shan Medical University, Taichung, Taiwan; 4 Cancer Research Center, Changhua Christian Hospital, Changhua, Taiwan; 5 Graduate Institute of Biomedical Sciences, China Medical University, Taichung, Taiwan; 6 Institute of Oral Sciences, Chung Shan Medical University, Taichung, Taiwan; 7 Department of Dentistry, Chung Shan Medical University Hospital, Taichung, Taiwan; 8 Whole-Genome Research Core Laboratory of Human Diseases, Chang Gung Memorial Hospital, Keelung, Taiwan; 9 Department of Medical Research, Chung Shan Medical University Hospital, Taichung, Taiwan; 10 Department of Biomedical Sciences, College of Medicine Sciences and Technology, Chung Shan Medical University, Taichung, Taiwan; 11 Division of Allergy, Department of Pediatrics, Chung-Shan Medical University Hospital, Taichung, Taiwan; IIT Research Institute, UNITED STATES

## Abstract

**Background:**

We identified *rs17071138 T/C*, *rs3744941 C/T*, and *rs8089104 T/C* gene polymorphisms of *SERPINB5 (mammary serine protease inhibitor)* that are specific to patients with oral cancer susceptibility and their clinicopathological status.

**Methodology/Principal findings:**

In total, 1342 participants, including 601 healthy controls and 741 patients with oral cancer, were recruited for this study. Allelic discrimination of *rs17071138 T/C*, *rs3744941 C/T*, and *rs8089104 T/C* of the *SERPINB5* gene was assessed by a real-time PCR with a TaqMan assay. We found that individuals carrying the polymorphic *rs17071138* and *rs8089104* are more susceptible to oral cancer (OR, 1.57; 95% CI, 1.07~2.31 and OR, 1.58; 95% CI, 1.04~2.39, respectively). Among oral cancer-related risk factor exposures, the individuals carrying the polymorphic *rs17071138* had 4.26- (95% CI: 1.65~11.01; *p* = 0.002), 2.34- (95% CI: 1.19~4.61; *p* = 0.01), and 2.34-fold (95% CI: 1.38~3.96; *p* = 0.001) higher risks of developing oral cancer.

**Conclusions:**

Heterozygous *TC* of the *SERPINB5 rs17071138* polymorphism may be a factor that increases susceptibility to oral cancer. Interactions of gene-to-gene and gene-to-oral cancer-related environmental risk factors have a synergetic effect that can further enhance oral cancer development.

## Introduction

SERPINB5 (a mammary serine protease inhibitor), a 42-kDa cytoplasmic protein that belongs to the serine family of protease inhibitors (serpins) is expressed in normal human mammary epithelial cells and has a protective role in the oncogenic process by inhibiting cell proliferation, development, invasion, angiogenesis, and metastasis [1–7].

Oral cancer is one of the most common malignancies in the world [8]. In Taiwan, it is the 6^th^ most prevalent malignancy [9] and the 5^th^ leading cause of cancer deaths (8.2/100,000) among Taiwanese [10]. Cellular senescence and apoptosis in oral cancer and oral precancerous cells are considered strong mechanisms for tumor suppression through their control of the proliferative potential of premalignant or malignant cells [11]. It was reported that repression of premalignant cell proliferation can be triggered by oncogene-induced senescence (OIS), and SERPINB5 was suggested to be an effector of oncogene-induced senescence, which acts as a natural barrier against transformation from a premalignant lesion to carcinoma in oral leukoplakia with dysplasia [12]. Gene expression of SERPINB5 was significantly higher in tissues of normal oral mucosa than in oral leukoplakic tissues with dysplasia and in oral squamous cell carcinoma tissues; however, there were no significantly different expression levels between tissues of normal oral mucosa and leukoplakia without dysplasia [12]. A loss of SERPINB5 expression was correlated with an increased invasive potential in a human oral squamous cell carcinoma (OSCC) cell line [1, 4]. Tumor cells lacking SERPINB5 expression tend to have lymph node metastasis and invasive progression of OSCC, and patients with high levels of SERPINB5 gene expression have better survival rates than those with low expression levels of SERPINB5 [1, 2, 4]. We suggest that SERPINB5 plays an important role in modulating the progression of oral cancer.

Single-nucleotide polymorphisms (SNPs) are reported to be important factors in oral cancer susceptibility [9, 13–15]. The *SERPINB5* gene is located on chromosome 18q21.3, and expression of the *SERPINB5* gene can be modulated by regulating the initiation of transcription on the promoter region in human oral cancer cell lines [16]. Two gene polymorphisms of *SERPINB5*, *rs17071138 T/C* and *rs3744941 C/T*, are found in the promoter region, and one SNP, viz., *SERPINB5 rs8089104 T/C*, is found in intron 1 [6, 17]. It was reported that *rs17071138 TC* SERPINB5 messenger (m)RNA expression in whole blood was significantly downregulated compared to the *rs17071138 TT* wild-type (WT) homozygous genotype [17]. rs3744941 can be attributed to the transcription regulator protein BACH2 (BTB domain and CNC homolog 2)-binding site [17]. Three SNPs, viz., *rs17071138 T/C*, *rs3744941 C/T*, and *rs8089104 T/C*, showed strong linkage to the *SERPINB5* promoter, which suggests that these three SNPs can reduce transcription factor-binding affinity with reduced SERPINB5 gene expression [17]. We suggest that these three SNPs can alter SERPINB5 protein levels, and considerably affect individual sensitivities to oral cancer. However, to the best of our knowledge, no studies have investigated the impacts of *SERPINB5 rs17071138 T/C*, *rs3744941 C/T*, and *rs8089104 T/C* gene polymorphisms on susceptibility to oral cancer. In this study, we recruited 1342 participants, including 741 patients with oral cancer and 601 healthy controls, to determine whether genetic variations in these regions of *SERPINB5* and their interactions with oral cancer-related risk factors are associated with susceptibility to and clinicopathological development of oral cancer.

## Materials and methods

### Subjects and specimen collection

In total, 741 patients who had been diagnosed with oral cancer according to characteristic criteria of national guidelines for oral cancer were recruited between April 2007 and April 2015 as a case group at Chung Shan Medical University Hospital in Taichung and Changhua Christian Hospital in Changhua, Taiwan. Meanwhile, 601 resident area-matched healthy individuals were randomly selected from the same geographic area, and these control groups had no self-reported history of cancer at any site. The study was performed with the approval of the Chung Shan Medical University Hospital (CSMUH) Institutional Review Board (no: CS13214-1), and informed written consent was obtained from each individual. Whole-blood specimens, collected from peripheral veins of recruited subjects, were placed in tubes containing EDTA, immediately centrifuged, and stored at -80°C.

### Genomic DNA extraction

We extracted genomic DNA from whole-blood samples using QIAamp DNA blood mini kits (Qiagen, Valencia, CA) according to the manufacturer’s instructions. DNA was dissolved in TE buffer [10 mM Tris (pH 7.8) and 1 mM EDTA] and then quantitated by measuring the absorbance at an optical density of 260 nm (OD_260_). The final preparation was stored at -20°C and used as templates in polymerase chain reactions (PCRs).

### Real-time PCR

Allelic discrimination of *rs17071138 T/C*, *rs3744941C/T*, and *rs80891041T/C* polymorphisms of the *SERPINB5* gene was carried out using the TaqMan SNP genotyping assay (Applied Biosystems, Foster City, CA). SERPINB5 rs17071138 (assay ID: C_33627662_20), rs3744941 (assay ID: C_27493638_10), and rs8089104 (assay ID: C_29202434_30) polymorphisms were assessed using an ABI StepOne™ Real-Time PCR System and analyzed using SDS vers. 3.0 software (Applied Biosystems). A genotyping fluorescence-based TaqMan SNP assay was used for the analysis. The final volume for each reaction was 10 μL and contained 5 μL of the TaqMan Universal PCR Master Mix, 0.25 μL of the primer/TaqMan probe mix, and 10 ng of genomic DNA. The real-time PCR consisted of an initial denaturation step at 95°C for 10 min followed by 40 cycles consisting of 92°C for 15 s and 60°C for 1 min [15, 17, 18]. The research assistant who conducted the laboratory analyses was blinded to the participants’ diagnoses.

### Sample size and statistical power

Based on the results of Kim et al. [6], assuming 95% confidence intervals (CIs) and *p*<0.05, our sample size had at least an 80% power to detect a 1.5-fold increased risk in susceptibility to oral cancer associated with the *SERPINB5 rs17071138*, *SERPINB5 rs3744941*, and *SERPINB5 rs8089104* genetic polymorphisms.

### Statistical analysis

Adjusted odds ratios (AORs) with their 95% confidence intervals (CIs) of the association of genotype frequencies with oral cancer risk and clinical characteristics were estimated by multiple logistic regression models after controlling for other covariates. A *p* value of <0.05 was considered significant. Data were analyzed with SAS statistical software (vers. 9.1, 2005; SAS Institute, Cary, NC).

## Results

This study estimated differences in demographic characteristics, such as gender, age, area of residence, race, and alcohol, tobacco, and areca (betel) nut consumption between oral cancer patients and controls. Significantly different distributions of gender, age, and alcohol, tobacco, and areca nut consumption between oral cancer patients and controls were found (data not shown). These variables with a statistically significant difference between oral cancer patients and controls were included in the multiple logistic regression models.

The individuals who are heterozygous for a *rs17071138 TC* polymorphism and with *CC* homozygotes of the *SERPINB5 rs8089104* polymorphism had 1.57- (95% CI: 1.07~2.31; *p* = 0.01) and 1.58-fold (95% CI: 1.04~2.39; *p* = 0.02) greater risks of developing oral cancer, respectively, compared to those with wild-type homozygotes of *SERPINB5 rs17071138 TT* and *SERPINB5 rs8089104 TT* after adjusting for confounding factors. A gene-to-gene interaction effect on the increased susceptibility to oral cancer was also found, the AORs and 95% CIs increased to 4.26- (95% CI = 1.74~10.40; *p* = 0.001) and 5.91-fold (95% CI = 2.14~16.35; *p* = 0.0006) greater risks of developing oral cancer for participants with at least one of the following, either *TC* or *CC* of *rs17071138*, *CT* or *TT* of *rs3744941*, or *CT* or *CC* of *rs8089104*, and for participants with *TC* or *CC* of *rs17071138*, and *CT* or *TT* of *rs3744941*, and *CT* or *CC* of *rs8089104* compared to participants with *TT* of *rs17071138*, *CC* of *rs3744941*, and *TT* of *rs8089104* (Table 1).

**Table 1:** Adjusted odds ratios (AORs) and 95% confidence intervals (CIs) of oral cancer associated with genotypic frequencies of *SERPINB5*.

Variable	Controls (*n* = 601) (%)	Patients (*n* = 741) (%)	AOR (95% CI)	*p* value
***SERPINB5 (rs17071138)***				
***TT***	485 (80.7%)	592 (79.9%)	1.00	
***TC***	109 (18.1%)	136 (18.3%)	1.57 (1.07~2.31)	*p* = 0.01
***CC***	7 (1.2%)	13 (1.8%)	1.38 (0.38~4.92)	*p* = 0.61
***SERPINB5 (rs3744941)***				
***CC***	248 (41.3%)	294 (39.7%)	1.00	
***CT***	276 (45.9%)	342 (46.1%)	1.00 (0.73~1.37)	*p* = 0.96
***TT***	77 (12.8%)	105 (14.2%)	0.76 (0.48~1.22)	*p* = 0.26
***SERPINB5 (rs8089104)***				
***TT***	152 (25.3%)	179 (24.2%)	1.00	
***CT***	281 (46.8%)	358 (48.3%)	1.44 (0.99~2.09)	*p* = 0.054
***CC***	168 (27.9%)	204 (27.5%)	1.58 (1.04~2.39)	*p* = 0.02
***SERPINB5* genes combination**				
Group 1	28 (4.7%)	11 (1.5%)	1.00	
Group 2	524 (87.2%)	671 (90.5%)	4.26 (1.74~10.40)	*p* = 0.001
Group 3	49 (8.1%)	59 (8.0%)	5.91 (2.14~16.35)	*p* = 0.0006

The AORs and 95% CIs were estimated by multiple logistic regression models, after controlling for gender, age, and alcohol, tobacco, and areca nut consumption. Group 1: individuals with *TT* of *rs17071138*, *CC* of *rs3744941*, and *TT* of *rs8089104*; Group 2: individuals with at least one of the following, *TC* or *CC* of *rs17071138*, *CT* or *TT* of *rs3744941*, or *CT* or *CC* of *rs8089104*; Group 3: individuals with *TC* or *CC* of *rs17071138*, *CT* or *TT* of *rs3744941*, and *CT* or *CC* of *rs8089104*.

Our study also determined whether there was an interactive effect of genes and relevant environmental risk factors on oral cancer susceptibility. The AORs and 95% CIs of genotypic frequencies and oral cancer susceptibilities were estimated among persons who were exposed to oral cancer-related environmental risk factors (Table 2). The individuals who are heterozygous for a TC polymorphism of the *SERPINB5 rs17071138* polymorphism had 4.26- (95% CI: 1.65~11.01; *p* = 0.002), 2.34- (95% CI: 1.19~4.61; *p* = 0.01), and 2.34-fold (95% CI: 1.38~3.96; *p* = 0.001) increased risks of developing oral cancer compared to those with WT homozygotes *TT* of *SERPINB5 rs17071138* among areca nut, alcohol, and tobacco consumers, respectively, after adjusting for confounding factors (Table 2). Moreover, we found that gene-gene interactions increased the risk of oral cancer susceptibility among subjects exposed to oral cancer-related risk factors, with AORs and 95% CIs increasing to 4.03- (95% CI = 1.36~11.95; *p* = 0.01), 7.93- (95% CI = 2.52~24.97; *p* = 0.0004), and 3.32-fold (95% CI = 1.15–9.55; p = 0.02) increased risks of developing oral cancer for participants with at least one of the following, *TC* or *CC* of *rs17071138*, or *CT* or *TT* of *rs3744941*, or *CT* or *CC* of *rs8089104*, and 10.61- (95% CI = 2.14~52.40.10; *p* = 0.003), 12.67- (95% CI = 3.12~51.43; *p* = 0.0004), and 5.02-fold (95% CI = 1.45~17.31; *p* = 0.01) increased risks of developing oral cancer for participants with *TC* or *CC* of *rs17071138*, and *CT* or *TT* of *rs3744941*, and *CT* or *CC* of *rs8089104* compared to participants with *TT* of *rs17071138*, *CC* of *rs3744941*, and *TT* of *rs8089104* when consuming areca nut, alcohol, and tobacco, respectively (Table 2).

**Table 2:** Adjusted odds ratios (AORs) and 95% confidence intervals (CIs) of oral cancer associated with genotypic frequencies of *SERPINB5* among individuals exposed to relevant environmental risk factors.

Variable	Controls	Patients	AOR (95% CI)	*p* value
**Among those who consumed areca nut (*n* = 679)**				
***SERPINB5 (rs17071138)***	Controls (*n* = 96) (%)	Cases (*n* = 583) (%)	AOR (95% CI)	*p* value
***TT***	88 (91.7%)	470 (80.6%)	1.00	
***TC***	5 (5.2%)	104 (17.8%)	4.26 (1.65~11.01)	*p* = 0.002
***CC***	3 (3.1%)	9 (1.6%)	0.46 (0.09-~2.24)	*p* = 0.33
***SERPINB5 (rs3744941)***				
***CC***	36 (37.5%)	225 (38.6%)	1.00	
***CT***	44 (45.8%)	268 (46.0%)	0.96 (0.57~1.59)	*p* = 0.87
***TT***	16 (16.7%)	90 (15.4%)	0.80 (0.40~1.59)	*p* = 0.53
***SERPINB5 (rs8089104)***				
***TT***	30 (31.3%)	150 (25.7%)	1.00	
***CT***	44 (45.8%)	284 (48.7%)	1.67 (0.97~2.88)	*p* = 0.06
***CC***	22 (22.9%)	149 (25.6%)	1.57 (0.83~2.98)	*p* = 0.16
***SERPINB5* gene combinations**				
Group 1	7 (7.3%)	10 (1.7%)	1.00	
Group 2	86 (89.6%)	529 (90.7%)	4.03 (1.36~11.95)	*p* = 0.01
Group 3	3 (3.1%)	44 (7.6%)	10.61 (2.14~52.40)	*p* = 0.003
**Among those who consumed alcohol (*n* = 640)**				
***SERPINB5 (rs17071138)***	Controls (*n* = 219) (%)	Cases (*n* = 421) (%)	AOR (95% CI)	*p* value
***TT***	185(84.5%)	334 (79.3%)	1.00	
***TC***	32 (14.6%)	81 (19.2%)	2.34 (1.19~4.61)	*p* = 0.01
***CC***	2 (0.9%)	6 (1.5%)	0.73 (0.10~5.19)	*p* = 0.75
***SERPINB5 (rs3744941)***				
***CC***	96 (43.8%)	174 (41.3%)	1.00	
***CT***	99 (45.2%)	189 (44.9%)	1.22 (0.74~2.01)	*p* = 0.42
***TT***	24 (11.0)	58 (13.8%)	1.40 (0.65~2.97)	*p* = 0.38
***SERPINB5 (rs8089104)***				
***TT***	46 (21.0%)	98 (23.3%)	1.00	
***CT***	103 (47.0%)	208 (49.4%)	1.18 (0.64~2.17)	*p* = 0.58
***CC***	70 (32.0%)	115 (27.3%)	0.98 (0.51~1.87)	*p* = 0.95
***CT or CC***	173 (79.0%)	323 (76.7%)	1.09 (0.62~1.93)	*p* = 0.74
***SERPINB5* gene combinations**				
Group 1	15 (6.9%)	7 (1.7%)	1.00	
Group 2	184 (84.0%)	376 (89.3%)	7.93 (2.52~24.97)	*p* = 0.0004
Group 3	20 (9.1%)	38 (9.0%)	12.67 (3.12~51.43)	*p* = 0.0004
**Among those who consumed tobacco (n = 861)**				
***SERPINB5 (rs17071138)***	Control (*n* = 227) (%)	Case (*n* = 634) (%)	AOR (95% CI)	*p* value
***TT***	190 (83.7%)	504 (79.5%)	1.00	
***TC***	36 (15.9%)	120 (18.9%)	2.34 (1.38~3.96)	*p* = 0.001
***CC***	1 (0.4%)	10 (1.6%)	2.01 (0.21~19.06)	*p* = 0.53
***SERPINB5 (rs3744941)***				
***CC***	99 (43.6%)	248 (39.1%)	1.00	
***CT***	96 (42.3%)	296 (46.7%)	1.09 (0.73~1.64)	*p* = 0.66
***TT***	32 (14.1%)	90 (14.2%)	0.76 (0.43~1.35)	*p* = 0.35
***SERPINB5 (rs8089104)***				
***TT***	54 (23.8%)	157 (24.8%)	1.00	
***CT***	104 (45.8%)	308 (48.6%)	1.50 (0.94~2.39)	*p* = 0.08
***CC***	69 (30.4%)	169 (26.6%)	1.40 (0.83~2.36)	*p* = 0.19
***SERPINB5* gene combinations**				
Group 1	7 (3.1%)	11 (1.8%)	1.00	
Group 2	201 (88.5%)	572 (90.2%)	3.32 (1.15~9.55)	*p* = 0.02
Group 3	19 (8.4%)	51 (8.0%)	5.02 (1.45~17.3%)	*p* = 0.01

The AORs and 95% CIs were estimated by multiple logistic regression models, after controlling for gender, age, and alcohol, tobacco, and areca nut consumption. Group 1: individuals with *TT* of *rs17071138*, *CC* of *rs3744941*, and *TT* of *rs8089104*; Group 2: individuals with at least one of the following, *TC* or *CC* of *rs17071138*, *CT* or *TT* of *rs3744941*, or *CT* or *CC* of *rs8089104*; Group 3: individuals with *TC* or *CC* of *rs17071138*, *CT* or *TT* of *rs3744941*, and *CT* or *CC* of *rs8089104*.

These genetic polymorphisms were analyzed in light of the clinical status of each of our recruited 741 oral cancer patients, including the tumor stage, tumor size, lymph node metastasis, distant metastasis, and cancer cell differentiation. There was no significant association of the clinical status with the *SERPINB5 rs17071138 T/C*, *rs3744941C/T*, or *rs8089104 T/C* gene polymorphisms in these patients (Table 3).

**Table 3:** Adjusted odds ratios (AORs) and 95% confidence intervals (CI) of clinical statuses associated with genotypic frequencies of *SERPINB5* in oral cancer patients (*n* = 741).

** Clinical stage**				
***SERPINB5 (rs17071138)***	Stage <II(*n* = 349) (%)	Stage ≥II(*n* = 392) (%)	AOR (95% CI)	*p* value
***TT***	278 (79.7%)	314 (80.1%)	1.00	
***TC***	63 (18.0%)	73 (18.6%)	0.99 (0.68~1.45)	*p* = 0.96
***CC***	8 (2.3%)	5 (1.3%)	0.57 (0.18~1.79)	*p* = 0.34
***TC or CC***	71 (20.3%)	78 (19.9%)	0.94 (0.65~1.35)	*p* = 0.76
***SERPINB5 (rs3744941)***				
***CC***	139 (39.8%)	155 (39.5%)	1.00	
***CT***	161 (46.1%)	181 (46.2%)	1.01 (0.73~1.38)	*p* = 0.95
***TT***	49 (14.1%)	56 (14.3%)	1.02 (0.65~1.60)	*p* = 0.92
***CT or TT***	210 (60.2%)	237 (60.5%)	1.01 (0.75~1.36)	*p* = 0.93
***SERPINB5 (rs8089104)***				
***TT***	85 (24.4%)	94 (24.0%)	1.00	
***CT***	170 (48.7%)	188 (47.9%)	0.99 (0.69~1.43)	*p* = 0.97
***CC***	94 (26.9%)	110 (28.1)	1.05 (0.70~1.58)	*p* = 0.80
***CT or CC***	264 (75.6%)	298 (76.0%)	1.01 (0.72~1.42)	*p* = 0.93
** Tumor size**				
***SERPINB5 (rs17071138)***	≤T2(*n* = 408) (%)	>T2(*n* = 333) (%)	AOR (95% CI)	p value
***TT***	325 (79.7%)	267 (80.2%)	1.00	
***TC***	77 (18.9%)	59 (17.7%)	0.94 (0.64~1.37)	*p* = 0.76
***CC***	6 (1.4%)	7 (2.1%)	1.39 (0.45~4.25)	*p* = 0.55
***TC or CC***	83 (20.3%)	66 (19.8%)	0.97 (0.67~1.40)	*p* = 0.89
***SERPINB5 (rs3744941)***				
***CC***	165 (40.4%)	129 (38.7%)	1.00	
***CT***	187 (45.8%)	155 (46.6%)	1.05 (0.76~1.44)	*p* = 0.75
***TT***	56 (13.8%)	49 (14.7%)	1.07 (0.68~1.68)	*p* = 0.76
***CT or TT***	243 (59.6%)	204 (61.3%)	1.05 (0.78~1.42)	*p* = 0.71
***SERPINB5 (rs8089104)***				
***TT***	103 (25.3%)	76 (22.8%)	1.00	
***CT***	198 (48.5%)	160 (48.1%)	1.14 (0.79~1.65)	*p* = 0.46
***CC***	107 (26.2%)	97 (29.1%)	1.29 (0.85~1.94)	*p* = 0.22
***CT or CC***				
** Lymph node metastasis**				
***SERPINB5 (rs17071138)***	No (*n* = 492) (%)	Yes (*n* = 249) (%)	AOR (95% CI)	*p* value
***TT***	396 (80.5%)	196 (78.7%)	1.00	
***TC***	89 (18.1%)	47 (18.9%)	1.02 (0.69~1.52)	*p* = 0.89
***CC***	7 (1.4%)	6 (2.4%)	1.86 (0.61~5.67)	*p* = 0.27
***TC or CC***	96 (19.5%)	53 (21.3%)	1.08 (0.74~1.58)	*p* = 0.67
***SERPINB5 (rs3744941)***				
***CC***	192 (39.0%)	102 (41.0%)	1.00	
***CT***	227 (46.2%)	115 (46.2%)	0.96 (0.68~1.33)	*p* = 0.80
***TT***	73 (14.8%)	32 (12.8%)	0.83 (0.51~1.35)	*p* = 0.46
***CT or TT***	300 (61.0%)	147 (59.0%)	0.92 (0.68~1.27)	*p* = 0.64
***SERPINB5 (rs8089104)***				
***TT***	119 (24.2%)	60 (24.1%)	1.00	
***CT***	232 (47.1%)	126 (50.6%)	1.05 (0.71~1.54)	*p* = 0.79
***CC***	141 (28.7%)	63 (25.3%)	0.86 (0.55~1.33)	*p* = 0.50
***CT or CC***	373 (75.8%)	189 (75.9%)	0.98 (0.68~1.40)	*p* = 0.91
** Distant metastasis**				
***SERPINB5 (rs17071138)***	No (*n* = 732) (%)	Yes (*n* = 9) (%)	AOR (95% CI)	*p* value
***TT***	584 (79.8%)	8 (88.9%)	1.00	
***TC***	135 (18.4%)	1 (11.1%)	0.49 (0.06~4.00)	*p* = 0.50
***CC***	13 (1.8%)	0 (0%)	―	*p* = 0.98
***TC or CC***	148 (20.2%)	1 (11.1%)	0.44 (0.05~3.64)	*p* = 0.45
***SERPINB5 (rs3744941)***				
***CC***	292 (39.9%)	2 (22.2%)	1.00	
***CT***	335 (45.8%)	7 (77.8%)	3.20 (0.65~15.80)	*p* = 0.15
***TT***	105 (14.3%)	0 (0%)	―	*p* = 0.97
***CT or TT***	440 (60.1%)	7 (77.8%)	2.49 (0.50~12.30)	*p* = 0.26
***SERPINB5 (rs8089104)***				
***TT***	177 (24.2%)	2 (22.2%)	1.00	
***CT***	352 (48.1%)	6 (66.7%)	1.38 (0.27~7.05)	*p* = 0.69
***CC***	203 (27.7%)	1 (11.1%)	0.36 (0.03~4.19)	*p* = 0.42
***CT or CC***	555 (75.8%)	7 (77.8%)	1.00 (0.20~4.97)	*p* = 0.99
**Cell differentiation grade**				
***SERPINB5 (rs17071138)***	Grade <II (*n* = 113) (%)	Grade ≥II (*n* = 628) (%)	AOR (95% CI)	*p* value
***TT***	90 (79.6%)	502 (79.9%)	1.00	
***TC***	21 (18.6%)	115 (18.3%)	0.97 (0.57~1.63)	*p* = 0.91
***CC***	2 (1.8%)	11 (1.8%)	0.97 (0.21~4.52)	*p* = 0.97
***TC or CC***	23 (20.4%)	126 (20.1%)	0.97 (0.59~1.60)	*p* = 0.91
***SERPINB5 (rs3744941)***				
***CC***	42 (37.2%)	252 (40.1%)	1.00	
***CT***	50 (44.2%)	292 (46.5%)	0.98 (0.62~1.53)	*p* = 0.93
***TT***	21 (18.6%)	84 (13.4%)	0.67 (0.37~1.21)	*p* = 0.19
***CT or TT***	71 (62.8%)	376 (59.9%)	0.89 (0.59~1.35)	*p* = 0.59
***SERPINB5 (rs8089104)***				
***TT***	30 (26.5%)	149 (23.7%)	1.00	
***CT***	56 (49.6%)	302 (48.1%)	1.05 (0.64~1.72)	*p* = 0.82
***CC***	27 (23.9%)	177 (28.2%)	1.26 (0.71~2.23)	*p* = 0.41
***CT or CC***	83 (73.5%)	479 (76.3%)	1.12 (0.71~1.78)	*p* = 0.61

The AORs and 95% CIs were estimated by multiple logistic regression models, after controlling for gender, age, and alcohol, tobacco, and areca nut consumption. >T2: multiple tumors of >2 cm in diameter. Cell differentiation grade: grade I, well differentiated; grade II, moderately differentiated; grade III, poorly differentiated.

## Discussion

To the best of our knowledge, this is the first study to provide novel information on the impacts of *SERPINB5 rs17071138 T/C*, *rs3744941 C/T*, and *rs8089104 T/C* genetic polymorphisms on the susceptibility to and clinicopathological development of oral cancer.

It was reported that genetic factors play important roles in oral cancer susceptibility, and discovery of potential oral cancer-related SNPs was recommended for the early detection of possible candidates for oral cancer development [9, 13–15]. SERPINB5 was reported to be a tumor suppressor by inducing apoptosis and inhibiting proliferation, invasion, and migration of tumor cells [19, 20]. No studies had examined associations of *SERPINB5 rs17071138 T/C*, *rs3744941 C/T*, and *rs8089104 T/C* genetic polymorphisms with susceptibility to oral cancer. Only two studies investigated the role of these three genetic polymorphisms in the susceptibility to gastric cancer [6] and hepatocellular carcinoma (HCC) [17]. Kim et al. [6] recruited 430 cases diagnosed as either diffuse-type gastric cancer (*n* = 252) or intestinal-type gastric cancer (*n* = 178) and 406 unaffected controls to examine relationships of the *SERPINB5 rs17071138 T/C*, *rs3744941C/T*, and *rs8089104 T/C* gene polymorphisms with susceptibility to gastric cancer. They found that none of the SNPs was associated with intestinal-type gastric cancer susceptibility; however, the presence of the *C* allele of *rs3744941 C/T* and the *T* allele of *rs8089104 T/C*, significantly increased the susceptibility to diffuse-type gastric cancer [6]. Yang et al. [17] enrolled 302 patients with HCC and 590 healthy controls to evaluate impacts of *SERPINB5* genetic polymorphisms on the HCC risk. They detected that the *SERPINB5 rs17071138 T/C*, *rs3744941 C/T*, and *rs8089104 T/C* genetic polymorphisms were not associated with HCC susceptibility [17].

In the present study, *TC* heterozygotes of the *SERPINB5 rs17071138 T/C* polymorphism and *CC* homozygotes of the *SERPINB5 rs8089104 T/C* polymorphism were significantly associated with susceptibility to oral cancer. Moreover, gene-to-gene interactions significantly increased the risk of oral cancer susceptibility among participants with *TC* or *CC* of *rs17071138*, *CT* or *TT* of *rs3744941*, and *CT* or *CC* of *rs8089104* polymorphisms of *SERPINB5*. It was demonstrated that oncogene-induced senescence contributed to repressing aberrant proliferation of oral squamous cells at risk of neoplastic transformation [11, 12]. SERPINB5 is a potential senescence marker in oral precancerous lesions for its response to oncogene-induced senescence, and increased SERPINB5 expression plays a critical role in protecting cells against oral cancer [1, 2, 4, 11, 12]. We suggest that genetic polymorphisms of *TC* heterozygotes of *rs17071138 T/C* and *CC* homozygotes of *SERPINB5 rs8089104 T/C* can decrease promoter activity resulting from alterations in transcription factor binding, which could diminish the production of SERPINB5 [17]. Such an occurrence of genetic variants or gene-gene interactions obstructing the senescence response for regulating cell cycle arrest and triggering cell apoptosis consequently induces transformation from precancerous cells to malignant cells and increases the susceptibility to oral cancer [1, 2, 4, 11, 12, 21, 22].

The individuals exposed to oral cancer-related environmental risk factors, including areca nut, alcohol, and tobacco consumption, were demonstrated to have increased risks of oral cancer by inducing mucosal fibroblast proliferation, oral epithelial hyperplasia, and dysplasia [23–29]. Moreover, alcohol consumption increased the malignant transformation of oral keratinocytes and oral cellular membrane penetration by carcinogens, thereby promoting the development of oral cancer [28, 30]. In our study, subjects with *TC* heterozygotes of the *SERPINB5 rs17071138* polymorphism and who were areca nut, alcohol, or tobacco consumers had respective 4.26-, 2.34-, and 2.34-fold significantly increased risks of developing oral cancer compared to those with WT *TT* homozygotes of *SERPINB5 rs17071138*, after adjusting for confounding factors. Moreover, gene-gene interactions increased risks of oral cancer susceptibility by 4.03-, 7.93-, and 3.32-fold among subjects exposed to oral cancer-related risk factors, for participants with at least one of the following, *TC* or *CC* of *rs17071138*, *CT* or *TT* of *rs3744941*, or *CT* or *CC* of *rs8089104* as well as increased risks of 10.61-, 12.67-, and 5.02-fold for participants with *TC* or *CC* of *rs17071138*, *CT* or *TT* of *rs3744941*, and *CT* or *CC* of *rs8089104* compared to participants with *TT* of *rs17071138*, *CC* of *rs3744941*, and *TT* of *rs8089104* when exposed to areca nut, alcohol, and tobacco consumption, respectively. Increased *SERPINB5* gene expression was reported to play an important role in protecting individuals from cancer development by inhibiting cell proliferation, development, invasion, angiogenesis, and metastasis [1–7]. Unfortunately, SERPINB5 expression in whole blood was significantly lower among individuals with the *rs17071138 TC* genotype compared to individuals with the *rs17071138 TT* WT homozygous genotype [17]. We suggest that individuals exposed to areca nut, alcohol, and tobacco risk factors might have increased susceptibility to oral cancer by the induction of oral epithelial hyperplasia and dysplasia [23–30]. Individuals with the *rs17071138 TT* WT homozygotes had higher SERPINB5 gene expression, which contributed to inhibition of oral cancer development; however, individuals with heterozygotes *TC* of the *SERPINB5 rs17071138* polymorphism exhibited decreased SERPINB5 protein function or expression, which contributed to a lack of response to oncogene-induced senescence and consequently promoted the development of oral cancer [17, 23–30]. In addition, gene-gene interactions decreased tumor suppression and consequently promoted the development of oral cancer, particular in subjects exposed to areca nut, alcohol, or tobacco consumption [17, 23–30].

In conclusion, our results suggested that heterozygotes *TC* of the *SERPINB5 rs17071138* polymorphism may be a factor that increases susceptibility to oral cancer. Interactions of genes and oral cancer-related environmental risk factors have a synergetic effect that can further enhance oral cancer development.
